# Murine Animal Models in Osteogenesis Imperfecta: The Quest for Improving the Quality of Life

**DOI:** 10.3390/ijms24010184

**Published:** 2022-12-22

**Authors:** Natividad Alcorta-Sevillano, Arantza Infante, Iratxe Macías, Clara I. Rodríguez

**Affiliations:** 1 Stem Cells and Cell Therapy Laboratory, Biocruces Bizkaia Health Research Institute, Cruces University Hospital, Plaza de Cruces S/N, 48903 Barakaldo, Spain; 2 Department of Cell Biology and Histology, University of Basque Country UPV/EHU, 48940 Leioa, Spain

**Keywords:** osteogenesis imperfecta, murine models, new therapies, preclinical studies, in vivo, bone, parameters, fragility

## Abstract

Osteogenesis imperfecta is a rare genetic disorder characterized by bone fragility, due to alterations in the type I collagen molecule. It is a very heterogeneous disease, both genetically and phenotypically, with a high variability of clinical phenotypes, ranging from mild to severe forms, the most extreme cases being perinatal lethal. There is no curative treatment for OI, and so great efforts are being made in order to develop effective therapies. In these attempts, the in vivo preclinical studies are of paramount importance; therefore, serious analysis is required to choose the right murine OI model able to emulate as closely as possible the disease of the target OI population. In this review, we summarize the features of OI murine models that have been used for preclinical studies until today, together with recently developed new murine models. The bone parameters that are usually evaluated in order to determine the relevance of new developing therapies are exposed, and finally, current and innovative therapeutic strategies attempts considered in murine OI models, along with their mechanism of action, are reviewed. This review aims to summarize the in vivo studies developed in murine models available in the field of OI to date, in order to help the scientific community choose the most accurate OI murine model when developing new therapeutic strategies capable of improving the quality of life.

## 1. Introduction

Osteogenesis imperfecta (OI), also known as “brittle bone disease”, is a rare genetic disorder that encompasses a group of conditions affecting the connective tissue. It is characterized by a decreased bone-mineral-density (BMD) alongside increased susceptibility to bone fractures, due to an abnormality in the synthesis and/or processing of the main protein of the bone extracellular matrix (ECM), the type I collagen molecule [1]. In approximately 85% of cases, it is caused by mutations in the *COL1A1* or *COL1A2* genes, encoding the α1 (I) and α2 (I) chains of type I collagen, respectively. In the remainder of the cases, mutations in up to 19 different genes related to type I collagen synthesis or processing have been identified up to the present date [2,3]. All these mutations contribute to two types of collagen I defects; quantitative (based on the reduction of type I collagen expression) and qualitative (structural alterations of the collagen I molecule). In addition to the genetic heterogeneity, OI exhibits clinical heterogeneity, mainly governed by the mutated gene, the type of mutation, the position of the mutation along the gene and the genetic background of the patient. Hence, genetic heterogeneity is translated into clinical phenotypes that range from mild (barely affected) associated with quantitative defects, to severe forms (qualitative ones), that in some cases (in the most severe phenotypes) result in perinatal mortality [4].

Nowadays, there is no curative treatment for OI [5]. The pharmacological treatments available have been extrapolated from osteoporosis, and focus on controlling the symptoms by increasing bones mass. In this respect, the most commonly used drugs are bisphosphonates (BPs), which target bone-resorbing cells (osteoclasts) [6]. BPs inhibit osteoclasts, decreasing the ratio of bone resorption and resulting in increased bone mass density. However, there are some concerns regarding their side effects, including a long half-life (which may even last for several years after discontinuing the treatment) and their scarce or limited effectiveness in some OI patients [7,8]. In this context, a monoclonal antibody (mAb) that inhibits osteoclast formation (Denosumab) has been positioned as a noteworthy alternative, since it has a relatively short degradation period, avoiding the long-term accumulation side effects of BPs. The first studies in patients with different types of OI have shown promising benefits, along with relatively high safety [9]. Moreover, a phase III clinical trial in postmenopausal women with osteoporosis, a reasonably prevalent low-bone-density condition, reported sustained elevation of BMD, together with low rates of adverse events and fracture incidence in patients, after 10 years of treatment with Denosumab [10]. Nevertheless, negative effects of Denosumab (rebound effects after stopping the treatment, hypercalcaemia, and hypercalciuria) have been reported, alerting the clinical community that further studies are demanded on this treatment [11,12]. 

On the other hand, further attempts are focused on stimulating bone formation, instead of inhibiting osteoclast function [13]. For instance, the synthetic parathyroid hormone (teriparatide) [14] has been proposed, but it has neither been used nor studied in children for the treatment of OI, because of concerns about potential osteosarcoma based on observations in animal studies [15]. Undoubtedly, there is an imperative demand for new drugs, with different mechanisms of action, leading to more effective and safer treatments able to be introduced into clinical practice. 

Among the crucial steps for developing efficient and safer treatments for OI, the in vivo studies in murine models are of a paramount importance. Given the high genetic and clinical heterogeneity of OI, it is crucial to identify the type of OI patients to be targeted, in order to select the most accurate murine model for the preclinical, in vivo studies. This means the best murine model available, which is competent to emulate the specific features of the OI type. Next, the efficacy of the treatment in vivo must be determined, based on clinically relevant bone parameters. Last but not least, the mechanism of action of the new therapeutic strategies proposed must be investigated. 

All in all, the present review will highlight the different murine OI models recently used in the search for OI therapies, then the bone parameters to be determined to evaluate the efficacy of different treatments in vivo, and lastly, the current and future therapeutic strategies for OI tested in the murine OI models, and their mechanism of action. 

## 2. Osteogenesis Imperfecta Murine Models Evaluated in Preclinical Research

In the quest for effective and safe therapeutic strategies for OI, there are a variety of mouse models available (although still insufficient), as expected and required for a disease as heterogeneous as OI. These murine models, carrying mutations in genes that encode collagen type I molecule, or molecules involved in collagen I processing, aim to emulate the genetic and phenotypic variability of OI patients. OI murine models encompass a wide range of severities depending on the mutation they carry, and although more than 20 different models for OI have been reported [16], in the present review only the most commonly used OI murine models in preclinical research are recapitulated. 

### 2.1. Osteogenesis Imperfecta Mice (oim)

The most commonly used OI murine model in preclinical studies is undoubtedly the osteogenesis imperfecta mice (oim). It was first described in 1993 by the Jackson Laboratory, when C3H/HeJ (a mice strain quite resistant to endotoxin) and C57BL/6JLe mice were bred, and a spontaneous mutation was generated [17]. Oim mice present a deficiency of the pro-α2 chain of the type I collagen molecule, due to a guanine (G) deletion at nucleotide 3983 of the *Col1a2* gene, which alters the approximately 50 terminal amino acids of the pro α2 C-propeptide. This mutation prevents association with the pro-α1 chain, resulting in tissue accumulation of aberrant αl homotrimeric collagen in the ECM. However, it has recently been reported that mice lacking the α2 chain of the type I collagen molecule, do not present impaired bone biomechanical or structural properties, unlike oim homozygous mice [18]. This observation emphasizes the oim mutant allele, and consequently its protein product, as the cause of the bone fragility seen in oim mice and not, as previously thought, the absence of the type I collagen α2 chain. 

The mutation that causes oim mice is very similar to the first identified mutation causing type III OI in humans, in which a 4 nucleotide deletion (c.4001_4004del) causes a frameshift (p.(Asn1334Serfs*34)) that alters the last 33 amino acids of the α2 chain of type I procollagen [19]. Moreover, novel mutations were identified in two additional patients with mild OI, causing a 48-amino-acid truncation of the α2 chain and a substitution of a cysteine residue important for interchain disulphide bonding, respectively [20]. In these cases, the mutations reported in humans also resulted in homotrimeric type I collagen synthesis, as the defective α2 (I) chain seemed to be unable to be incorporated into trimers [21]. Ultimately, homozygous oim mice resemble a severe, nonlethal, recessive form of human OI type III, presenting severe osteopenia, spontaneous fractures, small body size, and progressive skeletal deformities, along with very poor mechanical properties compared with their wild-type counterparts. These are features for which it is referenced as the only nonlethal murine model resembling the OI type III human disease.

Recent studies have focused on the understanding of different features of OI patients, extrapolating the results obtained with oim mice. As expected, the OI bone transcriptome of calvarial bone [22], together with the transcriptome of osteocytes isolated from the cortical femurs and tibias [23] of oim mice, was altered. I this process, genes involved in the Wingless-related-integration-site (Wnt) signaling and the transforming-growth-factor-beta (TGF-β) signaling were upregulated, compared to healthy counterpart animals. In terms of bone mineralization, oim bones are hypermineralized, which translates into a reduction in tissue elastic-modulus (the biomechanical parameter that measures stiffness). [24]. Recently, Zeng Z. and collaborators identified the deficient intrafibrillar mineralization in oim mice as a key contributor to OI-induced bone brittleness [25]. All in all, despite the fact that oim mice were spontaneously generated, and the mutation is not common in humans, they have proven to be undeniably useful, not only for studying collagen-matrix biology but for different preclinical studies in the context of type III OI. 

### 2.2. Heterozygotic G610C Mice (Amish Mice)

In 2010, Daley E. and colleagues reported a novel translational model for moderate OI, the G610C OI knock-in mouse. This mouse presents a substitution of the gly-610 codon for cysteine, in the protein encoded by the *Col1a2* gene, causing a final structure alteration of the type I collagen molecule. It is important to emphasize that the glycine substitution is the most common cause of dominant OI. In heterozygosis, it is also known as the Amish mouse, as it modeled a large human Amish population of 64 individuals exhibiting OI type I/IV, and is, together with oim, one of the most popular mice in OI-research labs [26]. Phenotypic characteristics of the mutant mice include decreased body weight, BMD, and bone volume, in addition to a more highly mineralized cortical and trabecular tissue, leading to decreased mechanical bone properties [27]. Cellular parameters have revealed that abnormally folded procollagen (due to glycine substitution) causes endoplasmic reticulum (ER) stress and osteoblast malfunction in Amish mice [28]. Indeed, misfolded procollagen in Amish osteoblasts indirectly triggers the integrated stress response (ISR) that is regulated, in this case, by the mitochondrial heat shock protein 70 and activating transcription factor 5 [29]. These outcomes suggest that mitochondria might initiate the ISR upon disruption of ER-mitochondria connections, or might respond to the ISR activated by a sensor as yet unknown. Amish mice also exhibit an impairment in the early phase of bone repair compared to wild-type littermates, due to an increased amount of type II collagen (typical of cartilage) to the detriment of type I (characteristic of bone) [30]. 

### 2.3. Brittle Mice (Brtl)

The Brittle IV (Brtl) mouse was developed as a knock-in model for OI type IV, by introducing a Gly349Cys substitution into one *Col1a1* allele, the glycine substitution again being the most common cause of dominant OI. Puzzlingly, in spite of having the same mutation, the mice showed phenotypic variability encompassing two different severities: moderately severe (resembling human OI Type IV) or perinatal lethal (resembling human OI Type II) [31]. Phenotypic variability is not associated with differences in expression levels of the mutant allele in the total ribonucleic acid (RNA) nor with the expression of the mutant protein. However, the different ability to adapt to cellular stress due to mutant collagen retention was found to be responsible for the heterogeneity of the Brtl mice phenotype. Thus, lethal mice showed a downregulation in the expression of cellular chaperones, as well as cytoskeletal disorganization in contrast with the moderately severe ones, who showed chaperone up-regulation and normal cytoskeleton [32,33]. Moderately severe Brtl mice were smaller in size and showed reduced BMD, reduced cortical thickness, reduced cross-sectional area and reduced biomechanical properties until 6 months of age [34].Two-month old Brtl mice showed higher skeletal fragility compared with wild type (WT) littermates, since the Brtl mutation alters the collagen orientation [35], affecting the mechanical behavior of bone material [36]. However, when Brtl mice reach puberty, they show an improvement in bone strength and stiffness, due to an increase in the material properties of the ECM [37]. All in all, Brtl mice reproduce the variability described in some OI patients, becoming an excellent model to study the factors that affect human OI phenotypes. In this case, autophagy upregulation has also been demonstrated in mutant mesenchymal-stem-cells (MSCs) differentiated toward osteogenic lineage, as a consequence of ER stress due to anomalous mutant-collagen retention [38].

### 2.4. Jrt Heterozygous Mice

Chen F. and collaborators identified a mouse manifesting dual features of type IV OI and Ehlers–Danlos syndrome (EDS), named Col1a1^Jrt/+^ (Jrt) mice [39]. EDS is a group of disorders that affect connective tissues supporting the skin, bones, blood vessels, and many other organs and tissues. Mutations in at least 20 genes (*COL5A1*, *COL5A2*, *COL1A1*, *TNXB*, *ADAMTS2*, *PLOD1* or *FKBP14*, among others) are associated with EDS. The combined phenotype presented relays on a mutation in a splice donor site of the *Col1a1* gene, which lead to the skipping of exon 9 and a consequent 18 amino-acid deletion within the amino (N)-terminal region of the triple helical domain of *Col1a1*. Jrt mice are smaller [40], have lower BMD, and worse trabecular microarchitecture, and therefore exhibit mechanically weak, brittle, fracture-prone bones, the hallmarks of OI. Additionally, they exhibit some characteristics of EDS, such as reduced tensile-properties of skin, a rayed tail tendon, and curvature of the spine.

### 2.5. Heterozygous Abnormal Gait 2 (Aga2) Mice

The murine model termed Aga2 (abnormal gait 2) described in 2008 [41] is the consequence of a carboxyl (C)-terminal frameshift mutation in the *col1a1* gene. Although the mutation affects *col1a1*, and up to the present date an equivalent genetic mutation has not been identified in humans, it is considered an appropriate model to emulate OI types II and III. Heterozygous Aga2 mice exhibit reduced bone mass, multiple fractures, and early lethality (due to cardiorespiratory defects) [42]. Moreover, they show increased bone turnover and a disrupted native collagen network, in which abnormal pro-α1 chains accumulated intracellularly in dermal fibroblasts are poorly secreted extracellularly. Thus, unfolded protein accumulation leads to an induction of an ER stress-specific unfolded protein response. 

### 2.6. Heterozygous Col1a1^±365^ OI Mouse

In order to emulate the OI type I phenotype, a heterozygous col1a1^±365^ OI mouse was newly generated by partial exons knockout (exon 2-exon 5; 365 nucleotides of mRNA) [43]. This deletion causes a frameshift mutation and premature chain termination, causing generally a large decrease in type I collagen synthesis. The OI murine model possesses significant femoral collagen reduction accompanied with sparse mineral scaffolds, bone loss, lowered mechanical strength and a broken bone-metabolism, pointing to a sustained skeletal weakness. The yes-associated protein (YAP), one of the key coactivators in the Hippo signaling pathway, known to play a pivotal role in osteoclasts and osteoblasts balancing in postnatal bone remodeling [44]) is altered in these mice. Hence, it is suggested that YAP down-regulation in both the femur and adipose-derived MSCs under osteogenic differentiation of col1a1^±365^ mice, may contribute to the reduced osteogenic potential and brittle bones. 

### 2.7. Crtap Mouse

Besides mutations in genes that encode type I collagen molecule (*COL1A1* and *COL1A2*), OI is also caused, to a lesser extent, by alterations in genes that encode proteins involved in type I collagen processing, among others. This is the case of the cartilage associated protein (CRTAP), which is a member of the prolyl 3-hydroxylation complex, crucial for post-translational modifications and functionalization of collagen molecules [45]. In humans, *CRTAP* mutations are associated with the clinical spectrum of recessive OI, ranging from neonatal lethal cases (OI type II) to a milder phenotype (OI type VII), depending upon the nature of mutation. Using a homologous recombination approach, a strain of homozygous Crtap-deficient (Crtap) mice has been produced, which seems to properly model OI VII in humans [46]. 

The loss of *Crtap* in mice causes an osteochondrodysplasia (inherited abnormalities of growth and development of connective tissue, bone, and/or cartilage), with rhizomelia (discrepancy of the length of the proximal limb), kyphosis (curvature of the spine, which causes the top of the back to appear more rounded than normal), as well as severe osteoporosis, with defective osteoid formation. Due to loss of *Crtap*, mutant collagen shows evidence of overmodification, presenting the collagen fibrils in mutant skin with increased diameter, consistent with altered fibrillogenesis. Crtap animals and OI type VII patients show an abnormally high mineral content and increased mineral densities in their bones [47]. The transcriptome of Crtap osteocytes have identified several dysregulated pathways already presented by other OI murine models, such as development and differentiation, ECM and collagen fibril organization, as well as Wnt and TGF-β signaling [23]. 

### 2.8. IFITM5 Transgenic Mice

The interferon induced transmembrane protein family 5 (Ifitm5), also known as the bone-restricted Ifitm-like protein, is known to regulate germ cell specification, its expression being prominent in osteoblasts. Humans with OI due to IFITM5 mutations mainly present a unique heterozygous replacement (c.-14C>T) of the 5′-untranslated region, which results in autosomal dominant OI type V. Accordingly, a transgenic mouse model with IFITM5 c.-14C>T mutation (IFITM5) exhibits severe skeletal malformation, as well as perinatal death [48,49]. Additionally, the limbs of the embryo exhibit a consistent delay in mineralization. Indeed, osteoblasts derived from embryo calvaria show decreased mineralization and reduced expression of osteoblast differentiation markers. 

To sum up, taking into account the wide phenotypic and genotypic variability that patients with OI present, the OI mice models available to use in preclinical studies are limited. Although they present a phenotype that closely simulates bone fragility, the mutation is not strictly the same in most cases, which could be a serious handicap when developing certain therapeutic strategies, depending on their mechanisms of actions. Nevertheless, in the last five years new murine models for OI have been established, which present different mutations from previously used models, in an effort to expand the genetic background of OI models and thus increasing the possibilities in the search for new therapies for OI: the novel seal mouse, presenting a decrease in Col1a1 mRNA expression and consequently type I collagen reduction, which in this case mimicks human type III OI [50]; homozygous lysyl hydroxylase 2 (LH2) mutant mice [51], resulting in embryos unable to develop normally that die at an early embryonic stage, due to cardiac problems; LH2 heterozygous mice, showing significant alterations in collagen crosslinking [52], as has been described in OI type XI patients [53]; the Swaying mouse model [54], presenting a spontaneous loss of function mutation in *Wnt1* and modeling OI type XV in humans [55]; the viable homozygous Wnt1^G177C/G177C^ mice [56]; the Ifitm5 S42L knock-in mouse model, resembling the atypical OI type VI [57]; and the Tent5a KO mouse, displaying bone fragility and a skeletal hypomineralization phenotype, as a result of quantitative and qualitative collagen defects [58]. Moreover, new OI models are being developed in different OI research groups, such as Brtl Ser (which presents the mutation G349S in the *col1a1* gene), high bone-mass mice (HBM; a mutation in the *col1a1* gene; type I procollagen c-propeptide cleavage defect), Tmem38b^−/−^ and Mbtps2. Finally, it remains to be demonstrated whether these new models are relevant to the search for new therapeutic strategies for OI.

The established OI mouse models already used in preclinical studies along with the new mentioned OI mouse models are summarized in Table 1. 

## 3. Revealing Parameters in Preclinical In Vivo Studies

**Figure 1:**
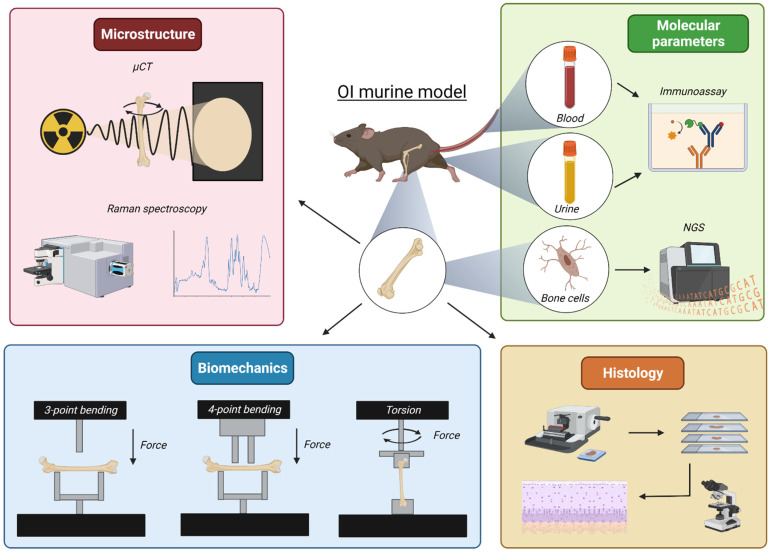
Bone properties evaluation. Different parameters are used to evaluate bone properties; histological and histomorphometric evaluation of bone; measurement of bone microstructure using image techniques (μCT and Raman spectroscopy), evaluation of biomechanics (3-/4-point bending test or torsion)) and analysis of molecular parameters (Immunoassay or NGS).

The main bone parameters that are considered when assessing potential treatments in OI murine models, and the different techniques that could facilitate the evaluation of the effects of these therapies, are discussed in the following lines (Figure 1). Histological and histomorphometric evaluation of bone are among the most popular and long-established determinations in the field. Later on, the advancement of computational techniques promoted the development of image processing software to automate bone histomorphometry analysis [59,60]. These techniques provide quantitative information about remodeling and bone structure using various staining and immunohistochemistry methods, allowing the identification of several markers such as osteoclast activity (tartrate-resistant acid phosphatase (TRAP) staining), alkaline phosphatase (ALP) activity, receptor activator of nuclear factor kappa-beta ligand (RANKL) or osteoprotegerin expression, among others [34,61]. In addition to histology and histomorphometry, the development of new technologies has allowed the implementation of wider range of techniques, most of them coming from the field of materials physics, which can offer additional molecular and structural information about organic and mineral bone tissue. 

### 3.1. Bone Microarchitecture

Bone tissue is composed of hierarchically organized materials that ultimately confer its mechanical properties and strength. Thus, bone fragility being the main feature of OI, it is expected that one of the most studied parameters is going to bone microarchitecture. This requires the use of spatial-resolution methods at different length scales to fully understand the underlying mechanisms that lead to altered physiological and functional properties of the tissue. In fact, several imaging techniques such as Raman imaging or mainly microcomputed tomography (µCT), have proven their value in the study of the bone structure for different OI mouse models. 

#### 3.1.1. Microcomputed Tomography

µCT, a powerful non-destructive imaging methodology based on X-ray radiation, produces high-resolution three-dimensional (3D) images composed of several two-dimensional (2D) projections of the studied object. Samples can be imaged with pixel sizes as small as 1 µm. Over the years, this technique has gain popularity, being the gold standard in studies requiring morphological and quantitative bone analysis, such as the assessment of animal models of disease [62,63,64]. In addition to 3D reconstructions, µCT allows the calculation of several trabecular and cortical parameters that provide information on the quality of the bone [65]. Trabecular bone parameters include bone volume-fraction (BV/TV), the number, thickness, and spacing of trabeculae (Tb.N, Tb.Th and Tb.Sp, respectively), BMD and connectivity density (Cn. Den). The cortical parameters include cortical thickness (Ct.Th), cortical bone area (Ct.Ar), tissue mineral density (TMD), cortical fraction area (Ct.Ar/Tt.Ar), cortical porosity (Ct. Por) and bone area per total area (BA/TA). 

#### 3.1.2. Raman Spectroscopy

Raman Spectroscopy is also a non-destructive technique which provides detailed information (micron scale) about chemical structure, phase transition (when a substance changes into a different state, e.g., from solid to liquid) and polymorphy (the existence of solid material in more than one structure), crystallinity and molecular interactions of the sample. Since bone ECM presents a highly crystalline structure, this tissue exhibits very strong Raman scattering (the inelastic scattering of protons by matter). Therefore, this technique can be extremely useful in the characterization of both mineral and organic matrix components of bone. Bone Raman spectrum gives information about several components of the bone, allowing the identification of collagen secondary-structure changes, proteins, immature and mature mineral bone, proteoglycans, and aminoacids (tyrosine, phenylalanine, proline, cysteine) [66].

### 3.2. Bone Biomechanical Properties

During normal activities, bone tissue is subjected to tensile, compressive, and shear stresses, causing a fracture when the bone tissue is subjected to these forces in excess of its strength. Of the several methods for measuring the effects of forces in bone, we will focus on the three main techniques. 

#### 3.2.1. Three- and Four-Point Bending

Three- and four-point bending, a structural mechanical test that measures the properties of the whole bone as a unique structure, tests the mechanical properties of the mid-diaphysis, which is typically all cortical bone. Therefore, it is appropriate for testing long bones such as the femur or tibiae, in which the obtained data is usually combined with the bone geometric properties obtained from µCT [63], to ultimately estimate bone material properties. Three-point bending produces its peak stress at the material mid-point and reduced stress elsewhere, while four-point bending produces peak stresses along an extended region of the material. These point-bending techniques give a plot of the load vs. displacement of the bone, describing its structural properties using five basic parameters: stiffness (the resistance of bone to displacement), yield load (the amount of load it can sustain before permanent damage), maximum load (also called ultimate force of strength), post-yield displacement (measuring ductility) and work-to-fracture (the work that must be done to fracture the bone). 

#### 3.2.2. Torsional Loading to Failure

The torsional loading-to-failure test is also designed to measure the strength of the sample, but in this case involves twisting the sample until it breaks. Torsional loading creates both transverse and longitudinal shear stresses in the bone, along with tensile and compressive stresses 45° from the shear direction. Ultimately, the bone will fail, creating a spiral fracture [67]. It is a useful test for acquiring several strength parameters such as torsional shear stress (the shear stress offered by the body against the torsional load), maximum torque (the maximum force applied at a distance that causes a change in angular momentum), shear modulus (the modulus of elasticity in shear, or the modulus of rigidity), torsional loading to failure (the amount of torsional load applied until fracture) and torsional ultimate strength (the maximum torsional stress-hold before rupture). 

### 3.3. Markers in Biological Samples: Blood and Urine 

There are several bone remodeling markers present in the biological fluids, able to correlate with the metabolic state of the bone; therefore they can be taken into consideration for disease and therapy monitoring. They can be divided into two main groups: bone-formation and bone-resorption markers. Bone-formation markers include osteocalcin (OST), ALP, N-terminal and C-terminal propeptide of type I procollagen (PINP and PICP, respectively). On the other hand, bone-resorption markers include N- and C-terminal cross-linking telopeptides of type I collagen (NTX and, CTX, respectively), pyridinoline and deoxypyridinoline cross-links (PYD and DPD, respectively), TRAP5b, bone sialoprotein (BSP) and Cathepsin K. All of these markers are usually measured using immunoassay techniques such as enzyme-linked immunoassay (ELISA), radioimmunoassay, and chemiluminescence, in addition to liquid chromatography—tandem mass spectrometry (LC–MS/MS) techniques. 

Although many of these bone-turnover markers can be analyzed both in urine and blood, the best source depends on the marker of interest [68]. Furthermore, some of these markers, such as CTX or OST, may vary with the circadian rhythm or diet. Thus, it is critical to take into account the peculiarities of each marker in order to minimize the biological variability and choose the most adequate type of sample (serum, plasma or urine), accordingly. Moreover, there are other parameters that can affect these markers, such as age and sex [69]. On the other hand, since bone remodeling is a complex biological process sustained by balanced osteoblast osteogenesis and osteoclast resorption, it is desirable to analyze more than one marker, in order to obtain a better overview of both processes [43,70]. 

### 3.4. Transcriptome Analysis

Transcriptome analysis permits the identification of genes that are differentially expressed between distinct conditions, leading to a deeper understanding of the genes and/or pathways related to those conditions. This knowledge is essential, not only when identifying potential therapeutic targets, but when evaluating the effects of new therapeutic attempts. In this context, RNA sequencing has revolutionized transcriptomics, thanks to the development of high-throughput next-generation sequencing (NGS). In fact, the transcriptomic analysis of different OI mice models has been crucial to better characterize the murine models and identify new altered signaling pathways. In this way, a transcriptomic study performed on the calvaria bone of oim and Jrt mice, showed that they shared 185 differentially expressed genes (106 upregulated and 79 genes downregulated in both models, with respect to their WT littermates). Among those shared 106 upregulated genes, several were involved in osteoblast function and ECM proteins, while others involved in Wnt and TGF-β signaling pathways were only upregulated in oim mice, but not, or to a lesser extent, in Jrt mice [22]. On the other hand, the study of the transcriptome of Crtap and oim osteocytes from femora and tibiae has shown similar alterations in both OI murine models, including type I collagen coding genes (*Col1a1* and *Col1a2*), bone-related transcripts *Bglap* and *Bglap2* (OST) and *Sparc* (osteonectin), and alterations in Wnt and TGF-β signaling pathways, suggesting a cellular attempt to increase osteoblast differentiation and function in response to pathogenic ECM [23].

## 4. Treatments for Osteogenesis Imperfecta: In Vivo Studies

**Figure 2:**
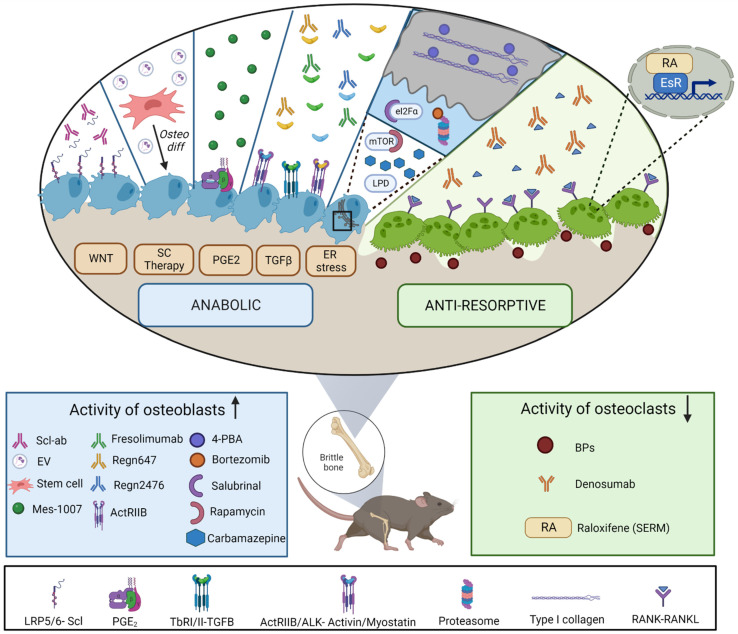
Treatments evaluated in OI preclinical studies to improve bone parameters of murine models. Anabolic therapies are illustrated in blue (targeting Wnt pathway, PGE2, TGFB superfamily, ER stress, or stem-cell therapy), while anti-resorptive ones are in green.

Several strategies have been studied in different OI murine models focusing on increase their BMD and improving their bone fragile phenotype, which can be divided in two different groups: antiresorptive therapies, aiming at decreasing or slowing down the resorption governed by osteoclasts, and anabolic ones, which try to increase the formation of bone by targeting osteoblasts (Figure 2). Table 2 summarizes in detail the established and newly attempted treatments mentioned below, together with their capacity to impact on crucial bone parameters, such as microstructure or biomechanics. 

### 4.1. Antiresorptives

Antiresorptive therapies, which inhibit the osteoclasts activity, including BPs, have been the most widely used therapies for OI until today. Simple BPs generate toxic adenosine triphosphate (ATP) metabolites while nitrogen-containing BPs act by inhibiting the enzyme farnesyl pyrophosphate (FPP) synthase, thereby preventing the prenylation of small guanosine triphosphatases that are necessary for the normal function and survival of osteoclasts [71]. Several OI mouse models treated with BPs, such as oim mice [72,73,74,75,76], and Brtl [77,78] have exposed, to a greater or lesser extent, the ability of BPs to increase bone density and reduce the average number of fractures [72]. Despite the encouraging preclinical studies and various clinical trials for patients with OI conducted (*NCT00159419*, *NCT00005901*, *NCT02303873*, *NCT00106028*, *NCT00131118*, *NCT00982124*), BPs present certain limitations, as mentioned before, such as side effects, a long half-life and a lack of efficiency in some OI patients.

The oim mice were again instrumental in evaluating the mAb therapy (Denosumab) focused on osteoclasts reduction by the inhibition of RANKL, exhibiting improved bone properties [79,80,81]. The related clinical trials performed (NCT01799798, NCT02352753, NCT03638128) demonstrated an increase in BMD [9], a normalization of vertebral shape, an increase of mobility, and a reduced fracture rate in patients with OI, after treatment with Denosumab [82]. Despite these encouraging outcomes, the clinical professionals are cautious regarding Denosumab due to some worrying effects reported, such as rebound effects after stopping the treatment, hypercalcemia, and hypercalciuria. 

Another FDA-approved antiresorptive therapy extrapolated from osteoporosis, centers on the use of a selective estrogen receptor modulator, known as Raloxifene, which has proven to reduce the incidence of fracture in oim mice [83]. In fact, its analog improved both trabecular and cortical microarchitecture and the mechanical properties of oim mice [84]. However, the associated side effects (such as hot flashes and increased thrombosis risk) prevent this hormonal estrogen therapy from being used in children with OI [84]. 

### 4.2. Anabolic Treatments

The anabolic strategies aim to avoid the adverse effects associated with anti-resorptive therapies, targeting bone-forming cells enhancing new bone generation.

#### 4.2.1. Targeting Wnt Signaling Pathway

When Wnt proteins bind to frizzled receptors and co-receptors of the low density lipoprotein receptor-related protein (LRP) family, β-catenin is stabilized, translocated into the nucleus, and activates the transcription of different genes, compromising MSCs differentiation into osteoblasts rather than chondrocytes or adipocytes [85,86]. The critical role of Wnt/β-catenin signaling pathway in regulating osteoblast differentiation and function accounts for it being the focus target of a wide variety of in vivo studies [87]. For instance, the role of a potent Wnt antagonist secreted by osteocytes, known as sclerostin, has been the focus of attention. Sclerostin binds to the Wnt co-receptors LRP5/6 and antagonizes downstream signaling [88,89]. Thus and as expected, mice lacking the gene that encodes sclerostin present a high-bone-mass phenotype [90], while overexpression of the same molecule decreases bone mass [91]. Encouraging preclinical studies have been carried out in several OI murine models able to mimic from a milder type of OI to the most severe ones (described below). Consequently, the anti-sclerostin antibody (Scl-Ab) has become the hub of intense clinical trials nowadays, treating OI patients by increasing osteoblasts activity.

Scl-Ab treatment has been evaluated in two mouse models of moderate OI presenting structural mutations in the type I collagen molecule, as a consequence of glycine substitutions in the *Col1a1* or *Col1a2* genes. Firstly, Scl-Ab successfully stimulated osteoblast bone formation, leading to improved trabecular and cortical bone-mass, while reducing long-bone fragility in rapidly growing 3-week-old [92], 8-week-old [93] and adult Brtl mice [94]. In fact, the study of bone quality of rapidly growing (3-week-old) and adult (6-month-old) Brtl animals (treated with Scl-Ab) rendered an increase in the mineral to matrix ratio [95], suggesting that Scl-Ab treatment positively alters the matrix chemistry of newly formed bone. In effect, these bone mass changes have been reported in some areas of the developing crania of Brtl mice [96]. The other moderate OI-murine-model evaluated, Amish mice, treated with Scl-Ab, provided similar results: increased bone mass and strength compared with vehicle-treated littermates [97]. Even more encouraging results were obtained when the therapeutic potential of the anabolic Scl-Ab was combined with the catabolic zoledronic-acid bisphosphonate, in comparison with either treatment alone. The combination of both drugs significantly enhanced BMD, cortical thickness and tissue mineral density, restoring the tibial strength in the Amish mice model of OI [98]. The studies on these OI moderate murine models highlight the potential of Scl-Ab therapy to increase bone formation without altering bone collagen composition, which may definitely benefit OI patients. 

On the other hand, preclinical studies on severe OI murine models treated with Scl-Ab have also been conducted, but with diverse outcomes. When Jrt mice, the mouse model of severe dominant OI, were treated, no effect was shown on weight no femur length, either in the serum markers of bone formation or resorption. The treatment was not able to improve either the microstructure or the biomechanical parameters of the murine bone [99]. On the contrary, when the severe recessive OI model, Crtap mice, were treated with the same molecule, bone phenotype was improved [100] in young and adult murine models. Hence, the Scl-Ab treatment led to an increase in bone volume and an improvement in trabecular and cortical microarchitecture in Crtap femurs in both age cohorts. In addition, biomechanical testing showed improved parameters of whole bone strength as a consequence of Scl-Ab treatment. 

Surprisingly, these studies did not provide any information on the number of fractures in Scl-Ab-treated mice. That information came from the most used mice (the model of severe type III OI), in particular the female oim mice, in which Scl-Ab significantly reduced long-bone fractures by improving bone mass, density, microstructure, and biomechanical strength [101]. Similar outcomes were observed when Scl-Ab was used to reduce vertebral fractures and spine deformities in oim mice [102]. Nevertheless, a different response was reported when male and female oim mice were treated with the mentioned antibody, characterized by the male-specific response of cortical bone to Scl-Ab and a lower number of fractures in the male [103]. In short, although Scl-Ab did not change the intrinsic properties of the OI bone matrix, it reduced the fracture rate of bones, supporting the potential of this antibody therapy in the reduction of bone fractures in certain OI severe patients. 

All in all, Scl-Ab has demonstrated promising beneficial effects in different murine models of OI, although not in all of them. Ultimately, the in vivo studies strongly suggest that its efficacy depends on animal age, tissue, length of treatment, genotype and gender. All these promising results reported in murine models of OI converged in a phase 2 clinical trial with Scl-Ab Setrusumab (NCT01417091), evidencing the stimulation of bone formation, a reduction in bone resorption, and an increase in lumbar spine BMD in Setrusumab-treated adults with moderate OI [104]. More clinical trials are being performed, in order to select a suitable dose of the antibody (NCT03118570) and to evaluate the effect on the reduction in total fracture rate (NCT05125809). Similarly, Romosozumab (which also selectively inhibits sclerostin) increases bone mass density and blood markers characteristic for bone formation in OI patients [105]. Romosozumab pharmacokinetics is currently under investigation in children and adolescents with OI (NCT04545554). 

#### 4.2.2. TGF-β Superfamily Modulators

The inhibition of TGF-β has emerged as a novel therapeutic avenue for OI patients, based on the increased activation of the TGF-β pathway found first in bone tissue of OI mice models and, more recently, in patients [106,107,108]. The Amish and Crtap mice were the first reported OI models showing an enhanced TGF-β signaling in their skeleton, whose modulation using the specific anti-mouse TGF-β neutralizing antibody (1D11) was able to rescue the phenotype, suggesting that this higher activation could play a role in the pathomechanisms of OI [106]. Later, a similar overactivation of the TGF-β pathway was also found in the calvarial bone tissue of the Jrt mice [107]. However, the concomitant inhibition of TGF-β signaling by the neutralizing antibody 1D11, did not elicit the same magnitude of beneficial outcomes, possibly due to the different extent of TGF-β pathway hyperactivation depending on the animal model. Thus, intraperitoneal 1D11 administration to 8-week-old female Crtap and Amish mice elicited the restoration of serum bone-turnover markers and significant improvements in bone microstructure and biomechanical parameters [106,109]. However, a similar treatment schedule in 8-week-old male Jrt mice did not show any recovery of bone-turnover markers, either in bone microstructure or in biomechanical parameters, although a slight increase in their femoral length was reported [107]. Interestingly, the same intervention in WT mice had a bigger effect on their bone mass, thus pointing to the fact that the effect of TGF-β pathway inhibition does not depend on increased TGF-β signaling [107]. The severity of the OI phenotype, which could lead to different amounts of active TGF-β in the bone matrix, has been pointed out to be a decisive factor in treatment effectiveness [107]. 

In fact, the clinical severity of OI, determined by the genotype, has also been suggested to be a key factor in the OI patients’ response to new therapeutic approaches, such as cell therapy (reported by our group) and anti-TGF-β [108,110,111]. Although the information regarding TGF-β pathway activation in patients is scarce, evidence points also to an increased TGF-β signaling in OI patients, especially in the most severe cases [108,111,112]. Thus, a severe OI pediatric patient has been shown to exhibit an increased expression and bioactivity of serum TGF-β compared to a moderate one, suggesting that higher anti-TGF-β doses could be needed in severe OI to achieve a clinical benefit [111]. This observation has also been recently reported in a phase I clinical trial (NCT03064074) targeting TGF-β by using an anti-human TGF-β mAb, fresolimumab, in eight adults with clinically moderate-to-severe OI. The patients received a single dose of either 1 mg/kg (four patients) or 4 mg/kg (four patients) of fresolimumab, and the safety and effectiveness of the therapy were evaluated 3 and/or 6 months after the treatment. Interestingly, only the moderately affected patients showed improvements in lumbar BMD. Thus, those moderate patients (n = 2) receiving the dose of 1 mg/kg showed robust increases in BMD 6 months after the treatment, whereas those receiving the dose of 4 mg/kg (n = 3) showed earlier improvements, at 3 months after the treatment, although intriguingly these were not maintained in the 6-month evaluation [108]. These encouraging results further support the assumption that the dose of TGF-β targeting strategies needed to obtain a clinical benefit would depend on the magnitude of TGF-β dysregulation.

The inhibition of other pathways encompassed within the TGF-β superfamily, such as the activin A signaling pathway, has also been addressed as a preclinical therapeutic strategy in OI mice models. Myostatin (also known as GDF-8) and activin A, mediate their signals through activin receptor type IIB (ActRIIB) and type I tyrosine kinase receptors ALK4, 5 or 7, eliciting a canonical signaling cascade via Smad proteins, which negatively affects skeletal muscle mass and bone remodeling in mice [113]. Subsequently, the targeting of this pathway has been found to protect against muscle and skeletal loss under several conditions, such as aging and microgravity leading to disuse atrophy [114,115]. In the context of OI, Jrt mice have been shown to exhibit higher activin A expression in serum and calvarial bone tissue, thus suggesting increased ActRIIB signaling in bone that could be targeted by specific therapies [116]. DiGirolamo and coworkers first addressed the inhibition of activin A signaling by using a ligand-trap approach. Thus, they administered soluble ActRIIB to 12-week-old oim mice as a decoy for myostatin and/or activin A, resulting in an increased muscle and trabecular bone mass that was not reflected in the biomechanical bone properties in treated animals, possibly due to the severe phenotype of the oim mice model. The effectiveness of an ActRIIB-based treatment was further confirmed in a later study performed on WT, Amish and oim mice [117]. Interestingly, improvements in trabecular bone microarchitecture were observed, regardless of the genotype, which was suggested to be a consequence of the observed increased number of osteoblasts and decreased number of osteoclasts elicited by the treatment in the femurs of mice. Moreover, improvements in cortical bone geometry were also reported in WT and Amish mice, consistent with the increased biomechanical strength also observed in these mice. Transcriptomic analysis of the tibiae of treated Amish male mice showed an increased expression of *Ctsk* (osteoclast marker) *Alpl, Dmp1* and *Phex* (osteoblast and osteocyte biomarkers), while female mice showed increased *Sp7* and *Serpinh1* (osteoblast markers), and *Csf1* (osteoclast marker) expression, relative to their vehicle-treated littermates [117]. Therefore, it was concluded that treatment effect was genotype- and gender-dependent. In line with this, 8-week-old Jrt mice, which exhibited increased activin A expression in serum and calvarial bone tissue, did not show trabecular bone phenotype improvements, either in microstructure parameters or in bone strength, after treatment with soluble ActRIIB [116]. However, they showed increased femoral length after treatment, a finding also reported in the Amish mice but not in the oim, suggesting a positive effect of the treatment on proliferating chondrocytes in the growth plate.

The elucidation of the molecular mechanism driving the beneficial effects in OI mice models treated with ActRIIB ligand traps has been hindered by the fact that they can bind to multiple ligands (myostatin, activin A, GDF-11 and some bone morphogenetic proteins). Moreover, there are different ActRIIB ligand traps, which vary in their sequence and therefore in their ligand binding [116]. A more recent strategy carried out by Charlotte Phillips‘s group tried to overcome this issue in OI mouse models, by specifically targeting myostatin with a humanized anti-myostatin mAb, Regn647 [118]. Surprisingly, only WT mice showed significant improvements in trabecular and cortical-bone parameters, as well as in femoral biomechanical strength, suggesting that the lack of response in the Amish mice could be due to the insufficient pharmacological inhibition of myostatin alone. This observation was later confirmed by the same authors, who addressed the inhibition of activin A (with Regn2476 antibody), myostatin, or both by using specific monoclonal antibodies in the WT and Amish mice [119]. Interestingly, the combinatorial inhibition of myostatin and activin A turned out to be considerably more efficient than myostatin or activin A inhibition alone, with the treated mice showing increased hindlimb muscle and body mass and improved bone microarchitecture and strength, regardless of sex and genotype [119]. These results highlight the implications of the functional redundancy between multiple ligands in the TGF-β superfamily, and open up the possibility of combinatorial strategies to target increased signaling of the TGF-β pathway in OI disease [115]. 

#### 4.2.3. Targeting Cellular Stress 

When the type I collagen molecule is synthetized, two α1 and one α2 chains of type I procollagen are assembled in the ER of the cells. However, severe OI patients present structural mutations (commonly Gly substitutions), preventing the appropriate binding among procollagen chains and leading to the misfolding of the type I collagen molecule. The misfolded collagen accumulates in the ER [120], causing ER dilation, cell stress and therefore dysfunction of osteoblasts in OI [28]. Indeed, and as expected, ER stress has also been reported in some animal models that mimic OI human mutations, such as Aga2 [41], Amish [28,121] and Brtl [38,122]. In order to relieve the pathogenicity caused by ER stress in OI patients, different pharmacological strategies have been proposed. These innovative attempts try to reduce cell stress by enhancing the clearance of unfolded collagen, and thus improving the dysfunction of osteoblasts in OI patients [123,124]. 

Among them, the chemical chaperone 4-phenylbutyrate (4-PBA) has shown promising results; it alleviates cellular stress (by increasing general cellular protein-secretion) and stimulates autophagy, in both dominant and recessive OI patients’ fibroblasts [124,125]. Similar results were obtained in vivo when appropriate OI mice models were used. Based on its molecular mechanisms, the chaperone would be expected to benefit OI patients presenting structural collagen mutations and thus cellular stress. In order to mimic the phenotype of these patients, Brtl and Amish mice were chosen, since both present glycine substitutions in the triple helical domain of type I collagen α1 and α2 chains, respectively. The stress alleviation reported an improvement of cell homeostasis in osteoblasts obtained from these two 4-PBA treated murine models, in addition to an increase in the quality of the OI ECMs [126]. Hence, the 4-PBA treatment for 4 weeks resulted in a significant improvement in the femur length and trabecular-bone parameters (analyzed using μCT) of the female Amish mice, compared with the PBS-treated controls, despite failing to improve cortical bone formation, geometry, or bone strength [127]. The efficacy of 4-PBA was also tested in the Aga2 model, with a higher dose than the one used in the Amish mice, and resulting in an increase in total body length and weight, decreased fracture incidence, increased femoral bone volume-fraction (BV/TV), and increased cortical thickness in both genders equally [128]. However, significant changes in the ultimate strength necessary to fracture were not observed in Aga2 mice treated with 4-PBA. In brief, 4-PBA treatment is able to reduce ER stress and improve OI bone quality in vivo in both the Amish and Aga2 mice. 

Moreover, different pharmacological molecules have been used for the purpose of alleviating ER stress. For instance, Brtl mice have been treated with the proteasome inhibitor Bortezomib, which ameliorated both osteoblast differentiation in vitro and bone properties in vivo [122]. Another therapeutic option focuses on salubrinal, an agent that reduces ER stress (by inhibiting the dephosphorylation of the eukaryotic-translation-initiation-factor 2 alpha), resulting in suppressed osteoclast maturation, along with the stimulated mineralization of MSCs. In fact, the in vivo studies carried out on oim mice subjected to 2 months’ salubrinal treatment rendered fairly promising outcomes, in which the femur-stiffness and elastic-module values matched those of the WT control [129]. 

Other strategies endeavored to alleviate ER stress by targeting the induction of autophagy, through pharmacological molecules or dietary control. For instance, in the rapamycin approach, rapamycin inhibits mTOR (the major regulator of growth that controls most anabolic and catabolic processes), which results in a retardation in protein synthesis and enhancement of cellular-stress-response pathways, such as autophagy. When the Amish mice were treated with rapamycin, an improvement in the trabecular parameters of the bone was shown, even though the biomechanical deficits of OI bones were not rescued [130]. On the contrary, deleterious effects were observed when rapamycin was directly administered to the E14.5 fetuses of the OI murine model resembling OI type V; the heterozygous Iftm5 transgenic mice with the c.-14C>T mutation. The treatment resulted in reduced whole skeletal bone-mineral-content (instead of a higher BMD), suggesting that rapamycin does not suppress the effect that causes the expression of mutated Iftm5 [131]. On the other hand, the carbamazepine treatment administered to stimulate the autophagy of misfolded collagen did not achieve the desired outcomes in the male Amish mice, being unable to improve either the bone microarchitecture or the biomechanics [132]. Last, but not least, diet has demonstrated its potential in the regulation of autophagy and thus in the evolution of OI. The degradation of misfolded collagen stimulated through autophagy in the Amish mice, by a low protein diet, resulted in osteoblast differentiation and bone-matrix mineralization, reducing overall procollagen synthesis [133]. However, a low-protein diet significantly suppressed overall animal growth in both the WT and Amish mice, as indicated by reduced weight gain and inhibited cortical drift, therefore preventing the reporting of an improvement in bone geometry and/or an increase in mechanical strength. 

#### 4.2.4. Prostaglandin E2 Receptor 

Prostaglandin E2 (PGE_2_) is known to increase bone remodeling, and can lead to substantial new bone formation in humans [134], EP4 being identified as the receptor that mediates bone formation in response to PGE_2_ [135,136]. Accordingly, further therapeutic attempts for OI are trying to promote bone formation by activating the EP4 receptor. However, as EP4 is widely expressed, EP4 agonists must specifically target the bone, to avoid potential adverse effects. In this context, a conjugate drug that consists of an EP4 agonist covalently linked to the BP alendronate (Mes-1007) has been developed. The effect of Mes-1007 treatment has been assessed in the Jrt severe-mouse-model of OI [137]. Despite the expectations, treatment with Mes-1007 alone did not lead to an improvement in trabecular-bone parameters in the distal femur or lumbar vertebra 4, either in the WT or the Jrt mice. A biomechanical test showed that WT femurs treated with a combination of Mes-1007 and the bisphosphonate zoledronate were stronger, but this result was not observed in the Jrt mice. 

#### 4.2.5. Stem-Cell-Therapy Approaches

Stem-cell therapy for treating OI, firstly addressed in murine models of the disease and later in patients, has been intensely studied for decades, with encouraging results. In fact, several clinical trials (summarized in Table 2) have addressed this approach in pediatric patients, with encouraging results. The rationale for using stem cells to treat OI was based on previous observations which reported that systemically infused MSCs in mice could repopulate both hematopoietic and non-hematopoietic tissues such lung, cartilage and bone [138,139]. Thus, Pereira and coworkers addressed the first approach, based on stem cell therapy in transgenic mice that carry a human mini-*COL1A1* gene, containing an internal deletion from intron 5 to intro 45, causing the synthesis of shortened proα1(I) chains [140]. The shortened proα1(I) chains bind to and produce the degradation of normal endogenous proα1(I) chains synthesized from the endogenous mouse *Col1α1* gene, leading to the development of OI bone phenotypes ranging from severe to mild, depending on the expression level of the mini-*COL1A1* gene. The therapy consisted of MSCs or whole bone marrow (BM) cells isolated from WT mice, which were intraperitoneally infused into 3-week-old irradiated OI-transgenic mice. One month after infusion, the bones of OI-transgenic mice receiving MSCs or BM showed a small but statistically significant increase in collagen and mineral content. Moreover, the authors demonstrated the donor-origin of a subset of cells in bone primary-cultures established 2–3 months after the cell therapy, which indicated donor cell engraftment in mice skeletal tissue [139]. 

After this turning point, a number of OI mice models resembling structural and quantitative defects in collagen type I were subjected to a myriad of cell therapy approaches with improvements in bone phenotypes, which were firstly attributed to the engraftment and differentiation of infused cells in bone tissue. Similarly, although first studies in mice reported improvements in OI-bone phenotypes after BM transplantation, the use of this cell source for OI-cell therapy should be treated with caution, in the light of recent results, reporting the absence of improvements in bone phenotypes after BM transplantation in oim and Amish mice models. This was linked to the finding that bone-associated donor cells were osteoclasts and osteal macrophages, rather than osteoblastic cells, pointing to a main engraftment and differentiation of hematopoietic progenitors and not of non-hematopoietic ones in host bone-tissue [141,142].

The reported low cell-engraftment in both animal models and patients [143,144] and the transitory nature of the clinical benefits, especially observed in patients, implied the existence of another unknown mechanism of cell therapy [110,144,145,146]. In fact, the existence of a synergy between the osteogenic differentiation of the scarce engrafted cells was confirmed, and the boosting of endogenous bone formation by the paracrine action of infused MSCs is more likely to account for the observed clinical benefits [110,147,148,149,150]. 

It is worth mentioning that, especially in the case of OI mice models, the stem-cell therapy attempts were carried out with substantial variation in the transplantation protocols. Thus, different delivery routes, time-points of administration, donor-cell sources and doses were tested, in an effort to find the most efficient experimental procedure. In spite of this high level of experimental variability, the great majority of experiments have revealed benefits in skeletal phenotypes after the cell therapy in OI mice models resembling both moderate-severe (structural-collagen-type-I defects) and mild (quantitative-collagen-type-I defects) OI.

##### Moderate-Severe OI Mice Models and Cell Approaches

Based on the number of published works, the oim mice have been the most used OI murine model for stem-cell therapy purposes. Initial works evaluated the feasibility of systemic transplantation of murine green-fluorescent-protein (GFP)^+^ allogenic MSCs (5 × 10^4^ cells) in sub-lethally-irradiated neonatal oim mice, and studied the in vivo distribution and engraftment of donor cells, as well as their collagen synthesis capacity [151,152]. Donor GFP^+^ cells, evaluated in bone chips from the femur and tibia of recipient mice, were shown to be engrafted at 4 weeks post-transplantation at variable levels, depending on the mice, ranging from 0.3% to 28%. Further histological analysis revealed that donor GFP^+^ cells colonized both the trabecular and cortical bone of recipient mice, and that they were co-distributed with the endogenous cells. An increased collagen staining in bones from treated mice compared to non-treated oim mice was also reported, suggesting than the engrafted cells synthesized and deposited normal collagen matrix in bone. 

After these studies, Guillot and coworkers intensively investigated the potential of transplanting fetal human MSCs in oim mice, with higher proliferative and osteogenic potential, due to their more primitive state [149,153,154,155,156]. First, they addressed the in utero transplantation (IUT) of human fetal blood MSCs (hfMSCs) isolated from fetuses in the first trimester of gestation, in oim embryos, thus providing a host-development environment promoting stem-cell expansion and engraftment. This prenatal administration of cells was also expected to prevent bone injury before irreparable impairment [153]. At 12 weeks postpartum, bones from mice subjected to IUT with hfMSCs showed a low bone-engraftment level (5%), which even so was higher than that shown by other tissues. Moreover, donor cells clustered in areas of high bone-remodeling, such as the growth plate and fracture sites. Noticeable improvements in mice skeletal phenotypes, such as a reduction in long-bone fractures and an increase in long-bone strength and cortical thickness were also shown, attributed to this cell engraftment as well as a possible paracrine mechanism elicited by the donor cells. These results were further confirmed in a later study, where the authors demonstrated that donor hfMSCs differentiated into mature osteoblasts that produced normal COL1A2, absent in nontransplanted oim mice, therefore producing normal collagen type I heterotrimers that modified the composition of the bone matrix to increase bone stiffness and decrease brittleness [154]. Later, the authors moved on to the intraperitoneal injections of human placental MSCs, a more available and ethical-concerns-free source of fetal cells, into oim neonates, with similar results [148,149,156].

As mentioned above, the low cell-engraftment (<2%) observed in patients and in OI mouse models after systemic cell infusion was surprising, based on the observed clinical benefits in both patient and animal models. Several strategies focused on improving the engraftment of MSCs have been addressed, encouraged by the fact that human mosaic carriers of OI mutations with just 25% of their osteoblasts producing normal collagen, show no skeletal phenotypes [157]. The low expression of key chemokine receptors promoting migration, such as CXCR4, has been suggested to account, at least in part, for this low engraftment of MSCs after systemic administration [155,158]. In order to overcome this issue, different approaches to upregulate the expression of CXCR4 have been assessed: For instance and in the case of OI mouse models, priming of hfMSCs with stromal cell-derived factor 1 (SDF1; the chemokine ligand of CXCR4) or with oim bone or plasma (both rich in SDF1) leaded to an upregulation of CXCR4 in hfMSCs, which in turn showed increased in vitro chemotaxis [155]. Moreover, in vivo experiments showed that intraperitoneal administration of 10^6^ primed hfMSCs in oim neonates elicited an increased cell-engraftment in bones, when compared with those transplanted with non-primed MSCs. This increased engraftment translated into a higher therapeutic potential of the cells, which elicited a greater decrease in fracture occurrence and improvements in bone mechanical properties and decrease in bone brittleness [155]. 

The systemic delivery of cells itself, with a significant proportion of MSCs becoming stacked in the lungs shortly after infusion, is thought to also be a factor hampering the migration of cells to the target sites [159]. A more recent cell-therapy approach performed in oim mice has addressed this issue by directly transplanting the MSCs into the femurs of oim mice [160]. The authors showed a higher proportion of engrafted cells (18%) using this delivery method, which differentiated into osteoblastic lineage and improved the bone mechanical properties of oim mice. Moreover, the infused MSCs were shown to be engrafted as long-term skeletal progenitors, since donor osteoblasts were observed 6 months post transplantation, thus exceeding the osteoblast life span of 14–60 days [160].

The minimal cell engraftment of cells when they are systemically infused appears to be insufficient to account for the observed beneficial effects in the skeleton of mice models and patients, suggesting that, in addition to differentiating into osteoblastic cells, MSCs provide trophic effects by secreting growth factors and cytokines that activate and support endogenous cells [149]. In fact, this hypothesis was confirmed in the Amish mice, who received extracellular vesicles (EVs) isolated from murine BM-MSCs, showing chondrocyte proliferation in the growth plate 8 weeks after the EVs treatment, which led to bone growth improvements in mice [150].

The efficacy of MSCs-based therapy has also been studied in other moderate-severe OI mice models although to a lesser extent than in oim mice. The only stem-cell approach tested in Brtl mice was an IUT of allogenic adult BM (5 × 10^6^ cells) from eGFP mice into embryos from WT females, crossed with Brtl males [161]. A low, variable, mice-dependent cell engraftment was reported in both hematopoietic and non-hematopoietic tissues from 2-month-old Brtl mice, including cortical and trabecular bone. As reported before, in the oim mice model, despite this low engraftment, skeletal improvements were observed in treated mice, such as a decreased fraction of mutant collagen, due to the presence of normal collagen synthesized by the engrafted cells and the restoration of the mechanical properties. Importantly, IUT completely prevented the perinatal lethality that a percentage of Brtl mice showed. The increased synthesis of normal collagen by engrafted donor cells, as well as the paracrine properties of these, were suggested to account for this unexpected result [161].

##### Mild-OI Mice Models and Cell Approaches

It is assumed that mild-OI patients affected by quantitative defects of collagen type I (OI Type I) could benefit from a cell therapy promoting bone formation. This is supported by the fact that MSCs would induce either directly, by engrafting in bone tissue, and/or indirectly, by paracrine mechanisms, the recovery of normal levels of collagen, which is otherwise structurally normal in these patients. 

Regarding OI mice models, this assumption has been tested in adult Col1a1^±365^ male mice. Thus, 4 weeks after receiving an intravenous injection of mice-adipose-derived MSCs, Col1a1^±365^ mice showed improvements in bone microstructure and increased bone formation [162]. Interestingly, in the same study, the authors showed that the administration of MSCs along with NELL1, an osteoinductive factor, achieved higher benefits than those shown by MSCs therapy or NELL1 alone. This result pointed to a synergistic effect between MSCs and and pro-osteogenic factors that opens up the possibility of combination therapy, in order to obtain better outcomes in OI patients. In addition, the therapeutic use of genetically corrected autologous stem-cell therapy has been tested in these mice, with encouraging results. Thus, whereas autologous adipose-derived MSCs from Col1a1^±365^ mice failed in restoring bone phenotypes because of the defective osteogenic-differentiation potential, corrected cells (rescued collagen-type-I expression) significantly improved the microstructure and mechanical properties of mice bones, by promoting bone formation [163]. This approach provides the proof of concept that genetically-modified MSCs could be used with therapeutic purposes for OI.

## 5. Discussion

There is a serious need for the development of safe and effective therapies for OI patients. Researchers in this field are making significant efforts to find effective therapeutic strategies able to at least improve the quality of life of the wide range of OI patients. With that goal in mind, bringing effective therapeutic strategies into the clinic, previous preclinical studies are mandatory, in which the murine models able to emulate different types of human disease are proving to be crucial tools. The present review summarizes the OI murine models available to date and the different therapies, as well as treatment attempts, addressed in them, attempting to guide the researchers in the selection of the OI murine model that best fits their goal, depending on the OI type, the potential of the specific model, and the target to be addressed.

In spite of the variety of animal models used up to the present time in in vivo OI studies, such as zebrafish (for OI type I-IV and OI type XIII), canine (OI type III golden retriever and beagle; OI type X dachshund) and mouse models [16], the latter are indisputably the most widely used, due to their relatively low cost, high rates of reproduction, and ease of handling and care, together with their ability to closely mimic the human disease. In fact, they present quite relevant similarities to humans in the following aspects: they have highly conserved physiological pathways, the same bone-cell and ossification types, the presence of long bones and BM hematopoiesis, age-related bone loss and a similar pattern of bone turnover and healing [164,165]. However, despite the multiple advantages of mice as in vivo models of OI, we cannot overlook the importance that zebrafish are gaining in the study of this disease, due to their short development-time, genetic similarity to humans, small size, low husbandry costs and embryo transparency [166]. In particular, the use of zebrafish has been strongly expanded for the investigation of human skeletal diseases [167], thanks to the possibility of easily generating mutant lines with the CRISPR/Cas 9 editing system [168], together with their ability to be a cheap tool for anabolic-bone-compound screening [169].

On the other hand, around 20 different types of OI murine models have been developed to date which present mutations in different genes and distinct phenotypes, aiming to cover the high heterogeneity seen in OI patients. The murine OI models already used in preclinical studies are well described and characterized. Efforts are being made to cover the variability seen in OI patients, from milder (col1a1^±365^ OI mouse) to more severe (Jrt, oim mouse) forms. In fact, the OI murine models developed mimic OI type I (col1a1^±365^/Amish), II (Aga2), III (Aga2/oim/Seal), IV (Amish/Brtl/Jrt), V (IFITM5), VI atypical (Ifitm5 p.S42L) VII (Crtap), XI (LH2 mutant), XV (Wnt ^G177C/G177C^) and XVIII (Tent5a KO), among others. Notwithstanding their ability to emulate the different phenotypes presented by patients, they present limitations that need to be taken into consideration. For instance, in the case of oim mice, although they are widely used in preclinical studies, the mutation is not very common in OI patients; in fact, the model was spontaneously generated. Conversely, the Amish and Brtl mice present a glycine substitution in *col1a1/2* genes, which is one of the most common mutations in dominant OI patients. Moreover, it is important to highlight the fact that not all the OI murine models are commercially available today, which hinders their used by the scientific community in the development and evaluation of new therapeutic attempts.

On the other hand, the fundamental role that the developed OI murine models have played in increasing knowledge of the disease and its etiopathology is undoubted. In this way, ER stress was reported in mice with structural mutations in the collagen molecule (such as Brtl, Amish and Aga2), due to the accumulation of abnormally folded collagen; the altered organization of collagen fibrils was studied, and the importance of post-translational modifications was demonstrated, such as procollagen proline 3-hydroxylation in Crtap mouse. In addition, other tissues besides bone are affected in the OI murine models. Oim mice present circumferential breaking strength and greater compliance of aortae [170], reduced ultimate stress and strain for tendons [171], skeletal muscle weakness [172] and kidney glomerulopathy [173]. Some of these features, such as alterations in aortae [105], tendons [174] and muscle [175], have been previously described in patients with OI.

Murine models have also been crucial in highlighting additional distinctions to be taken into consideration, such as the age- and gender-related differences [176] reported in some OI murine models, such as oim mice [103,177]. These gender-related differences are also reflected when the mice are subjected to different therapies, such as the male-specific response to Scl-Ab [103], and the different transcriptomic expression of ActRIIB-based treatment in female and males [117]. Therefore, it would be more than advisable to treat male and female mice separately, and to take this into account for translation to the patient.

Considering the limitations that anti-resorptive therapies present, this review has focused mainly io the anabolic ones, which induce bone formation by targeting signaling pathways altered in OI (TGF-β superfamily or ER stress) or by inducing osteoblast differentiation and activity (cellular therapies or the Wnt signaling pathway). Occasionally, a combination of two different treatments has been assessed, such as the growth hormone together with zoledronic-acid treatment, synergistically improving the microstructure, but not the biomechanical properties, of treated OI mice [178]. In addition, new types of dual therapies could be extrapolated from osteoporosis, such as the monoclonal Scl-Ab Romosozumab, which stimulates bone formation and inhibits resorption [105]. The stem-cell therapies tested and validated in OI mice models have culminated in the realization of early-phase clinical trials in OI pediatric patients, rendering promising results [110,144,145,146]. Of special interest is the TERCELOI clinical trial, the first study testing the safety and potential of the consecutive administration of five infusions of HLA-matched allogenic BM-MSCs in two non-immunosuppressed OI pediatric patients (moderate and severe OI). First, no adverse effects were reported, demonstrating the safety of the treatment. During the clinical trial, the patients showed noticeable improvements in bone phenotypes, such as a decrease in the number of fractures and enhancements of bone parameters. Moreover, the patients’ quality of life also improved during the course of the clinical trial. TERCELOI was also the first study to assess the molecular response of OI patients to cell therapy, finding a systemic pro-osteogenic paracrine response as a consequence of MSCs infusions [110]. Currently, there is an ongoing European phase I/II multicenter trial (BOOSTB4) assessing the safety, tolerability and efficacy of prenatal and postnatal doses of fetal allogeneic MSCs for severe OI patients carrying classic glycine substitutions [179]. Undoubtedly, the outcomes of all these early-phase clinical trials will pave the way for the development of an advanced therapy based on MSCs for OI in the not-too-distant future.

Another important point to be considered is that not all treatment attempts have demonstrated their effectiveness in the murine models evaluated, highlighting once again the heterogeneity of the disease and the critical choice of the precise type of patient (and the most accurate murine model) for whom a given treatment will be effective, emphasizing so-called precision medicine. For instance, when the TGF-β signaling pathway and modulators were targeted (with 1D11 antibody and soluble ActRIIB), different responses were reported in the oim, Amish, Crtap and Jrt OI murine models [107,117], due to their underlying bone phenotype, which in turn is caused by different mutations leading to different collagen type I defects with distinct severity. In this regard, none of the therapies in general have shown promising results in the Jrt mice model, suggesting that the severity of the OI disease and the underlying collagen defect could be playing a role in the diminished response to treatment. Finally, some of the therapies show only improvements in the bone microstructure and not in the biomechanical parameters [128], highlighting the importance of measuring different parameters when evaluating a new therapeutic attempt.

All in all, this review summarizes the preclinical murine models used at present for the development of therapies in OI, their characteristics, the bone-parameters evaluated, and the different outcomes, highlighting their limitations and potentials; these are variables to be taken into consideration for future safe and effective treatment-development able to improve OI patients’ quality of life.

## Figures and Tables

**Table 1:** OI murine models already used in preclinical studies. Models mimicking different types of OI. In all cases, similar mutations are reported in humans, except in the case of knockout mutations.

OI Mouse Models Used in Preclinical Studies				
Type OI In Humans	Mouse Model	Mutated Gene	Mutation	Consequences
III	oim	*Col1a2*	3983delG	Accumulation of αl homotrimeric collagen I
I/IV	Amish mice	*Col1a2*	610G>C	Structural: ER stress + osteblast malfunction
IV	Brtl	*Col1a1*	349G>C	Structural
IV + EDS	Jrt	*Col1a1*	exon9del	Quantitative: collagen I ↓
III/II	Aga2	*Col1a1*	C-terminal fsX	ER stress
I	Col1a1^±365^	*Col1a1*	exon 2-exon 5del	Quantitative: collagen I ↓
VII	CRTAP	*Crtap*	KO	
V	IFITM5	*Ifitm5*	14C>T	

**Table 2:** Treatments evaluated in preclinical studies with OI murine models. Parameters that were improved (**✓**) or not (**✗**) under different therapy attempts in the reported OI murine models are summarized. Moreover, therapies that have been addressed in clinical trials with OI patients are exposed.

Type of Treatment	Target	Treatment	Bone Parameters	oim	Amish	Brtl	Jrt	Aga2	Col1a1^±365^	crtap	Ifitm5	Clinical Trials
Anti-resorptive	ATP-dependent enzymes/FPP synthase	BPs	MST	**✓**		**✓**						NCT00159419, NCT00005901, NCT02303873, NCT00106028, NCT00131118,
			BIOM	**✓**		**✓**						
	RANKL	Denosumab	MST	**✓**								NCT01799798, NCT02352753, NCT03638128
			BIOM	**✓**								
	SERM	Raloxifene	MST	**✓**								
			BIOM	**✓**								
Anabolic	Wnt pathway	Scl-Ab	MST	**✓**	**✓**	**✓**	**✗**			**✓**		NCT01417091, NCT03118570 NCT05125809, NCT04545554
			BIOM	**✓**	**✓**	**✓**	**✗**			**✓**		
	TGF-β superfamily	α-TGF-β Ab	MST		**✓**		**✗**			**✓**		NCT03064074
			BIOM		**✓**		**✗**			**✓**		
		ActRIIB	MST	**✓**	**✓**		**✗**					
			BIOM	**✗**	**✓**		**✗**					
		α–myost Ab	MST		**✗**							
			BIOM		**✗**							
		α–activin A + α–myost Ab	MST		**✓**							
			BIOM		**✓**							
	Cellular Stress	4 PBA	MST		**✓**			**✓**				
			BIOM		**✗**			**✗**				
		Bortezomib	MST			**✓**						
			BIOM									
Anabolic + Anti-resorptive		Salubrinal	MST	**✗**								
			BIOM	**✓**								
Anabolic		Rapamycin	MST		**✓**							
			BIOM		**✗**							
			BMC								**✓**	
		Carbamazepine	MST		**✗**							
			BIOM		**✗**							
		Low protein diet	MST		**✗**							
			BIOM		**✗**							
	PGE2	Mes-1007	MST	*			**✗**					
			BIOM	*			**✗**					
	SC Therapy	MSCs	MST	**✓**					**✓**			NCT03706482, NCT04623606, NCT01061099, NCT05559801, NCT02172885
			BIOM	**✓**					**✓**			
		EVs-MSCs	BG		**✓**							NCT00705120, NCT00187018
		BM	MST	**✗**		**✓**						
			BIOM	**✗**		**✓**						

* Ongoing; MST: microstructure; BIOM: biomechanics; BG: bone growth; BMC: bone mineral content; SERM: selective estrogen receptor modulators; BM: bone marrow.

## Data Availability

Not applicable.

## Abbreviations

**Abbreviation**	**Definition**
µCT	Microcomputed tomography
2D	Two-dimensional
3D	Three-dimensional
4-PBA	4-phenylbutyrate
N-terminal	Amino- terminal
ActRIIB	Activin receptor type IIB
ATP	Adenosine triphosphate
BM	Bone Marrow
BMD	Bone mineral density
BPs	Bisphosphonates
C-terminal	Carboxyl- terminal
CTX	Carboxyl-terminal cross-linking telopeptide of type I collagen
ECM	Extracellular matrix
ELISA	Enzyme-linked immunoassay
ER	Endoplasmic reticulum
EVs	Extracellular vesicles
FPP	Farnesyl Pyrophosphate
GFP	Green fluorescent protein
hfMSCs	Human fetal blood MSCs
ISR	Integrated stress response
IUT	In utero transplantation
LH2	Lysyl hydroxylase 2
LRP	Low density lipoprotein receptor-related protein
mAb	Monoclonal Antibody
MSCs	Mesenchymal stem cells
NGS	Next-generation sequencing
OI	Osteogenesis Imperfecta
OST	Osteocalcin
PGE_2_	Prostaglandin E2
RANKL	Receptor activator of nuclear factor kappa beta ligand
RNA	Ribonucleic acid
Scl-Ab	Anti-sclerostin antibody
SDF1	Stromal cell-derived factor 1
TGF-β	Transforming growth factor beta
TRAP	Tartrate-resistant acid phosphatase
WNT	Wingless-related integration site
WT	Wild type
YAP	Yes-associated protein
